# Research on Path Planning Method for Mobile Platforms Based on Hybrid Swarm Intelligence Algorithms in Multi-Dimensional Environments

**DOI:** 10.3390/biomimetics10080503

**Published:** 2025-08-01

**Authors:** Shuai Wang, Yifan Zhu, Yuhong Du, Ming Yang

**Affiliations:** 1 School of Mechanical and Automotive Engineering, Liaocheng University, Liaocheng 252000, China; shuaiwang@lcu.edu.cn; 2 College of Management and Economics, Tianjin University, Tianjin 300072, China; yfzu@tju.edu.cn; 3 Key Laboratory of Advanced Mechatronics Equipment Technology, Tiangong University, Tianjn 300387, China; 4 School of Mechanical Engineering, Tiangong University, Tianjin 300387, China; 5 Analysis & Testing Center, Beihang University, Beijing 100191, China; myang_16@buaa.edu.cn

**Keywords:** optimized search algorithm, adaptive balance, search performance evaluation, path planning applications

## Abstract

Traditional algorithms such as Dijkstra and APF rely on complete environmental information for path planning, which results in numerous constraints during modeling. This not only increases the complexity of the algorithms but also reduces the efficiency and reliability of the planning. Swarm intelligence algorithms possess strong data processing and search capabilities, enabling them to efficiently solve path planning problems in different environments and generate approximately optimal paths. However, swarm intelligence algorithms suffer from issues like premature convergence and a tendency to fall into local optima during the search process. Thus, an improved Artificial Bee Colony-Beetle Antennae Search (IABCBAS) algorithm is proposed. Firstly, Tent chaos and non-uniform variation are introduced into the bee algorithm to enhance population diversity and spatial searchability. Secondly, the stochastic reverse learning mechanism and greedy strategy are incorporated into the beetle antennae search algorithm to improve direction-finding ability and the capacity to escape local optima, respectively. Finally, the weights of the two algorithms are adaptively adjusted to balance global search and local refinement. Results of experiments using nine benchmark functions and four comparative algorithms show that the improved algorithm exhibits superior path point search performance and high stability in both high- and low-dimensional environments, as well as in unimodal and multimodal environments. Ablation experiment results indicate that the optimization strategies introduced in the algorithm effectively improve convergence accuracy and speed during path planning. Results of the path planning experiments show that compared with the comparison algorithms, the average path planning distance of the improved algorithm is reduced by 23.83% in the 2D multi-obstacle environment, and the average planning time is shortened by 27.97% in the 3D surface environment. The improvement in path planning efficiency makes this algorithm of certain value in engineering applications.

## 1. Introduction

Path planning is a key technology for mobile platforms to realize intelligent functions such as environmental sensing and autonomous operation. Algorithms are utilized to enable mobile platforms to find optimal feasible paths from the initial point to the target location in different spaces, thereby improving operational efficiency [1]. Path planning algorithms can be broadly categorized into two classes: traditional algorithms and bionic intelligence-based algorithms [2], and they are widely used in fields such as electric power inspection [3], underwater search [4], and military task assignment [5]. Traditional algorithms can be subdivided into global and local path planning algorithms according to the utilization degree of environmental information. Global path planning provides approximate paths, while local path planning makes corrections within a local range of the global path to adapt to environmental changes and special needs. The global path planning algorithm needs to consider obstacle information in the environmental map, with representative algorithms including the A* and RRT algorithms [6]; the local path planning algorithm needs to update real-time environmental information via sensors, with representative algorithms such as the artificial potential field method and D* algorithm [7]. With the development of artificial intelligence technologies, bionic swarm intelligence algorithms that simulate biological evolution, group social behavior, and information exchange in nature have been formulated using mathematical expressions [8]. Early representative algorithms of swarm intelligence algorithms are particle swarm optimization (PSO) and ant colony optimization (ACO) [9]; medium-term representative algorithms are artificial bee colony (ABC) and artificial fish swarm algorithm (AFSA) [10]; and recent representative algorithms are sparrow search algorithm (SSA), whale optimization algorithm (WOA), and beetle antennae search (BAS), etc. [11]. The swarm intelligence algorithms have independent solution thinking and they can be adjusted adaptively and iteratively according to the path planning characteristics and requirements. Rich population categories help to achieve parallel computing and improve search efficiency while also having excellent scalability for larger-scale and more complex scenarios [12].

Swarm intelligence algorithms include two phases: global exploration and local optimization. The former performs a highly stochastic global search, while the latter performs deep optimization as the number of iterations in the search area increases. Regulating the balance between these two phases is particularly important. If the weight in the early exploration stage is too large, the algorithm may generate an unordered search, which slows down the convergence speed and wastes computing resources. If the weight in the later stage is too large, the algorithm may fall into local optimal oscillations and be unable to escape extreme points in the later stage [13]. The swarm intelligence algorithms do not rely on the initial parameter settings, and they are more inclined to explore the environmental information with a wider search range. The beetle algorithm has a high degree of structuring. It can not only simulate an individual beetle to judge the information concentration in the environment and solve problems independently but also be converted into a population of beetles to establish information interaction for a common solution. Some scholars combined the powerful search ability of the swarm algorithm with the low complexity of the beetle algorithm to improve the global search ability and local planning speed of the algorithm [14]. Cui et al. [15] introduced globally optimal individuals into both the employed bees and scout bees stages and introduced the teaching- and learning-based optimization algorithm into the peak-peak stage to enhance the algorithm’s mining ability and searching efficiency in terms of swarm population. Ni et al. [16] proposed a multi-strategy integrated bee colony algorithm, which utilizes different individual experiences with multiple search strategies to update the global path. Li [17] and Comert [18] combined the bee colony algorithm with the ant colony algorithm to improve the quality of the initial solution, optimize the number of path turns, and avoid blind searches. Liang et al. [19] added a virtual goal to the global search points to achieve fast local search optimization. Yu et al. [20] divided the global search process into several segments using the water flow potential field method and optimized the local search coordinate points according to the obstacles’ characteristics using the beetle algorithm to prevent falling into local optimal points. In addition, introducing chaotic functions and reverse learning mechanisms are the main strategies for improving the performance of swarm intelligence algorithms. Chen et al. [21] and Geng et al. [22] incorporated non-uniform variation and chaotic mapping functions into the sparrow search algorithm, respectively, which enhances the randomness and traversal ability of the populations. Mohapatra et al. [23] introduced a stochastic reverse learning mechanism into the golden jackal algorithm to overcome the limitations of balancing exploration and exploitation and improve the convergence speed. Hu et al. [24] introduced variational and greedy strategies into the rabbit algorithm to improve the quality of spatial solution exploration and population exploitation. The aforementioned literature has improved the efficiency of algorithms in path planning by means of hybrid swarm intelligence algorithms, introducing optimization strategies, etc. However, there are still problems such as early convergence of global exploration, low local optimization accuracy, and high dependence on parameter settings.

According to the No Free Lunch Theorem [25], no single intelligent optimization algorithm can universally solve all path planning problems. Even a well-optimized single algorithm fails to simultaneously address the dual challenges of global search and local optimization. To tackle this, the IABCBAS algorithm is tailored for path planning in multi-dimensional environments. This algorithm leverages swarm intelligence mechanisms for global exploration of path waypoints while employing the beetle algorithm for local path refinement. The main contributions of this study are summarized as follows.

Firstly, Tent chaotic perturbation is employed to enhance bee population diversity. By non-uniformly adjusting the updated swarm positions, the algorithm broadens the distribution of solution sets, optimizes search point quality, and boosts convergence speed. Secondly, a random reversal strategy is adopted to increase search direction points, thereby expanding the optimization range of the beetle algorithm and strengthening its ability to escape local optima. Position-based greedy selection is employed to achieve higher convergence accuracy. Thirdly, an adaptive inertia weight factor is introduced to autonomously adjust the weight distribution between the two algorithms, thereby achieving a balanced trade-off between global search capability and local optimization performance. Fourthly, path planning simulation experiments are conducted; the swarm algorithm performs global path pre-search, while the beetle algorithm handles local optimization. Two-dimensional, three-dimensional, and real-world environments are established to verify the engineering application value of the proposed algorithm.

This paper is structured into five parts. Section 1 provides a relevant introduction to the research content. Section 2 elaborates on the artificial bee colony algorithm, beetle swarm algorithm, and their respective improvement strategies. Section 3 focuses on testing the performance of the proposed algorithm. Section 4 presents path planning experiments conducted in 2D, 3D simulation environments, and a real-world environment. Section 5 includes conclusions and future prospects.

## 2. Hybrid Path Planning Based on Improved ABC and BAS Algorithms

### 2.1. ABC Algorithm for Global Path Exploration Optimization

#### 2.1.1. The Optimized ABC Algorithm

The ABC algorithm model is optimized as an “employed–onlooker–scout” model, which makes full use of the contribution of each type of bee in the global nectar search process and determines the location of the next path point based on the fitness value. Given that the ABC algorithm excessively relies on the optimization capability of leading bees, it tends to bypass the exploration of fitness values in unknown regions by scout bees, which leads to stagnation in the later stages of the search. To address this, the Tent chaotic perturbation mechanism and non-uniform mutation operator are introduced, respectively, aiming to enrich population diversity while enhancing the exploration capability of the search space, thereby generating more global waypoints available for selection by the algorithm.

Assuming there are *A* bees in a B-dimensional search space, the position of each bee is shown as

(1) $$C=\left[\begin{array}{cccccc} c_{1,1} & c_{1,2} & \ldots & c_{1,b} & \ldots & c_{1,B}\\ c_{2,1} & c_{2,2} & \ldots & c_{2,b} & \ldots & c_{2,B}\\ \vdots & \vdots & \ddots & \vdots & ⋰ & \vdots \\ c_{d,1} & c_{d,2} & \ldots & c_{d,b} & \ldots & c_{d,B}\\ \vdots & \vdots & ⋰ & \vdots & \ddots & \vdots \\ c_{A,1} & c_{A,2} & \ldots & c_{A,b} & \ldots & c_{A,B} \end{array}\right],$$

where d=1,2,…,A; b=1,2,…,B; and $c_{d,b}$ is the position of the *d*-th bee in dimension ***B***.

The employed bees guide the movement of the entire honey colony and can search for nectar sources anywhere in the region. Their updated position is shown in

(2) $$c_{d,b}^{e+1}=\left\{\begin{array}{l} c_{d,b}^{e}\exp\left(\frac{-b}{\alpha \cdot {\mathrm{iter}}_{\max}}\right),0.5\leq A\leq 1\\ c_{d,b}^{e}+FG,0\leq A<0.5 \end{array}\right.,$$

where *e* is the number of model iterations; ${iter}_{max}$ is the maximum number of iterations; α is a random number with 0<α≤1; *F* is a random value obeying the normal distribution function; and ***G*** is a 1×b matrix where each element is 1. A∈[0,1], which denotes the concentration of nectar in the region. When 0<A<0.5, the current concentration of nectar in the region is low, and the search range needs to be expanded. When A≥0.5, the current concentration of nectar is high, and all the honeybees need to fly to the region quickly.

The onlooker bees follow the employed bees in search of nectar and may harvest nectar together with the employed bees to enhance the nectar concentration of the entire population. Their updated position is shown in

(3) $$c_{d,b}^{e+1}=\left\{\begin{array}{l} F\exp\left(\frac{c{\mathrm{worse}}_{b}^{e}-c_{d,b}^{e}}{d^{2}}\right),d>\frac{A}{2}\\ c{\mathrm{best}}_{b}^{e+1}+\frac{1}{B}\sum_{b=1}^{B}\left(\left|c_{d,b}^{e}-c{\mathrm{best}}_{b}^{e+1}\right|•\mathrm{rand}\left\{-1,1\right\}\right),d\leq \frac{A}{2} \end{array}\right.,$$

where $c{worst}_{b}^{e}$ is the global worst position of the bee in the *b*-th dimension at the *e*th iteration; $c{best}_{b}^{e+1}$ is the global optimal position of the employed bee in the *b*-th dimension at the (*e* + 1)-th iteration; and *rand* is a randomly assigned value within the given range. When d>A/2, it means that the *d*th onlooker bee, which has low adaptability, is more likely to fail to collect honey due to the low nectar concentration in the region. Otherwise, the *d*-th onlooker bee finds a random position near the optimal nectar source where the employed bee is located to collect honey.

The random initial position of the scout bees is shown in

(4) $$c_{d,b}^{e+1}=\left\{\begin{array}{l} c{\mathrm{best}}_{b}^{e}+\beta \left|c_{d,b}^{e}-{\mathrm{cbest}}_{b}^{e}\right|,k_{d}\neq k_{b}\\ c_{d,b}^{e}+J\left(\frac{c_{d,b}^{e}-c{\mathrm{worse}}_{b}^{e}}{\left|k_{d}-k_{w}\right|+\varepsilon }\right),k_{d}=k_{b} \end{array}\right.,$$

where β denotes the step control parameter in the model, following a random normal distribution with a mean of 0 and variance of 1; *J* is a random value between $\left[-1,1\right]$; $k_{d}$ is the current fitness value of the bees; $k_{b}$ is the current optimal fitness value in the global search; $k_{w}$ is the worst fitness value; and ε is a non-zero minimum constant. $k_{d}\neq k_{b}$ ndicates that the scout bee is located at the edge of the population and $k_{d}=k_{b}$ indicates that the scout bee has found a better source of nectar and needs to move to a new region.

#### 2.1.2. Improvement Strategies

The Tent chaotic function [26] is adopted to enrich the population diversity, prevent the ABC algorithm from falling into a premature convergence situation during the optimization process, and improve the algorithm’s global search capability. The chaotic mapping function has strong traversal and randomness and can be mathematically mapped to transform chaotic sequences into individual spatial search capabilities. Among them, Tent chaos has a faster iteration speed and better uniformity, but there may be errors on immovable points. Therefore, random variables are introduced into the Tent chaos function to control the random values within a certain range and keep the algorithm’s regularity. Firstly, during the initialization of the bee population, a random initial individual is generated as a b-dimensional vector, where each dimension is within the interval [0,1]. Subsequently, the next individual is generated by substituting the value of each dimension of the current individual into the Tent chaotic formula sequentially to update the corresponding dimension of the new individual. This process of generating new individuals is repeated until (*n* − 1) new individuals are produced. All generated individuals are then mapped to the variable range, resulting in the Tent chaotic initial bee population. The initial population is generated as

(5) $$z_{d+1}=\left\{\begin{array}{l} \frac{2z_{d}+\mathrm{rand}\left(0,1\right)}{N},0\leq z_{d}\leq \frac{1}{2}\\ \frac{2\left(1-z_{d}\right)+\mathrm{rand}\left(0,1\right)}{N},\frac{1}{2}<z_{d}\leq 1 \end{array}\right.,$$

where *N* is the number of particles within the sequence, and rand (0,1) takes a random number in that range.

A non-uniform mutation operator is introduced to enhance solution accuracy. The non-uniform mutation operator [27] can introduce large perturbations in the early iterations of the algorithm, thereby enhancing the exploration of the search space. In the later stages, it reduces the perturbation magnitude, promoting the algorithm’s convergence to the vicinity of the global optimal solution and improving the quality of the global solution. For each bee generation after position update, the *d*-dimensional vector is randomly perturbed using a variance probability $l_{m}$, which decreases dynamically with the number of iterations. The mutation formulas for the *o*-th dimensional component are shown in

(6) $$c_{d}^{o+1}=\left\{\begin{array}{l} c_{d}^{o}+\Delta \left(e,p_{o}-c_{d}^{o}\right),\alpha =0\\ c_{d}^{o}-\Delta \left(e,c_{d}^{o}-q_{o}\right),\alpha =1 \end{array}\right.,$$

(7) $$\left\{\begin{array}{l} \Delta \left(o,r\right)=r\times \left(1-s^{{l_{m}}^{t}}\right)\\ l_{m}=1-\frac{e}{E_{\max}} \end{array}\right.,$$

where $p_{0}$ and $q_{0}$ are the maximum and minimum values of the vector range, respectively; α randomly takes the value 0 or 1; *e* is the current number of iterations; $E_{max}$ is the maximum number of iterations; *s* is the number of random numbers in the interval [0,1]; and *t* determines the dependence degree of random number perturbation on the iteration count *e*. In the early stage of the algorithm, *e* is small, and the operator performs uniform search in the space. In the later stage, as *e* increases, the new solution generated by mutation infinitely approximates the optimal real solution, yielding the best nectar source.

### 2.2. Local Path Optimal Optimization Based on BAS Algorithm

#### 2.2.1. The Optimized BAS Algorithm

The BAS algorithm is a novel heuristic algorithm based on simulating the mate and foraging behavior of beetles. If the ABC algorithm generates an excessive number of global waypoints, the BAS algorithm, with its strong local search capability, can obtain multiple locally optimal waypoints. This algorithm determines the optimal solution by comparing the fitness values of the current and the next waypoints. In this section, improvements are made to the optimization direction and fitness value confirmation of the BAS algorithm. The introduction of random reverse learning and a greedy selection mechanism enhances the reliability of the algorithm’s waypoint selection and improves the accuracy of waypoint optimization. The beetles’ search direction vector $\vec{d}$ is defined using the random search formula

(8) $$\vec{d}=\left|\frac{\mathrm{rand}\left(b,1\right)}{\left\|\mathrm{rand}\left(b,1\right)\right\|}\right|,$$

where *b* is the spatial dimension.

The initial position of the beetle in the *b*-dimensional space is shown as follows:

(9) $$c^{0}=(c_{1}^{0},c_{2}^{0},\cdot \cdot \cdot ,c_{b}^{0}).$$

At the *e*-th iteration, the beetle’s tentacles determine the left and right directions at the current position as follows:

(10) $$\left\{\begin{array}{l} c_{1}^{e}=c^{e}+\frac{c_{e}^{d}}{2\vec{d}}\\ c_{r}^{e}=c^{e}-\frac{c_{e}^{d}}{2\vec{d}}\\ c_{e,d}^{d+1}=0.95c_{e}^{d}+0.01 \end{array}\right.,$$

where $c_{e}^{d}$ denotes the distance between the two tentacles of the beetle at the *e*-th iteration; and $c_{e}^{d+1}$ is the updated length of the tentacles.

The beetle position of the next iteration is updated as

(11) $$\left\{\begin{array}{l} c^{e+1}=c^{e}+{\mathrm{step}}^{e}\vec{d}\mathrm{sign}[k(c_{1}^{e})-k(c_{r}^{e})]\\ {\mathrm{step}}^{e+1}=\mu {\mathrm{step}}^{e} \end{array}\right.,$$

where ${step}^{e}$ denotes the iterative step size of the beetle in the *e*-th iteration, which determines the convergence speed of the algorithm’s optimization search; μ is the step factor; $sign\left(\cdot \right)$ is the sign function; and $k(c_{l}^{e})$ and $k(c_{r}^{e})$ denote the objective function values corresponding to the left and right directions at the *e*-th iteration, respectively. As the number of iterations increases, $k(c_{r}^{e})$ continuously updates the convergence value until the optimal objective function solution is obtained.

#### 2.2.2. The Improvement Strategy

The beetle algorithm, characterized by few parameters and a simple structure, tends to steer randomly in local paths. Therefore, positional stochastic inverse learning and a greedy selection strategy [28] are introduced to optimize the judgment of left and right directions. Currently, the beetle’s direction selection is limited to left and right, with the remaining position information being underutilized. A local search in nearby directions is required to find a better position and avoid random steering. The stochastic inverse learning method generates a stochastic inverse solution based on the left and right directions, thereby increasing direction options and avoiding blind movement, as shown in

(12) $$c_{\mathrm{new}}^{e}=c_{1}^{e}+c_{r}^{e}-c_{\mathrm{rand}}\times c_{\mathrm{best}}^{e},$$

where $c_{new}^{e}$ denotes the newly generated random direction and $c_{best}^{e}$ is the current optimal position.

Since the fitness value of the generated new position may be lower than those of the left and right directions, a greedy selection of the beetle’s direction is performed to expand the search range and avoid local optima. In each iteration, the fitness values of the generated new position direction and the original left and right directions are compared. If the fitness value of the new direction is better than those of the original left and right directions, the beetle moves toward the new direction for updating as per Equation (12); otherwise, it moves as Equation (11). The next optimal position of the beetle is obtained through iterative screening using this method, as shown in

(13) $${\mathrm{kc}}^{e+1}=\left\{\begin{array}{l} k(c_{\mathrm{new}}^{e}),k(c_{\mathrm{new}}^{e})>k(c_{1}^{e})-k(c_{r}^{e})\\ k(c_{1}^{e}),k(c_{1}^{e})-k(c_{r}^{e})>k(c_{\mathrm{best}}^{e})\\ k(c_{r}^{e}),k(c_{1}^{e})-k(c_{r}^{e})<k(c_{\mathrm{best}}^{e}) \end{array}\right..$$

### 2.3. Adaptive Adjustment to Algorithm

In the early stage of the improved algorithm, the number of individual bees in the population is large and scattered. Adaptively adjusting the ABC algorithm to obtain a larger weight value can accelerate the convergence process and enhance the ability to explore global waypoints. As the number of iterations increases, the weight of the ABC algorithm gradually decreases, while the weight of the BAS algorithm gradually increases. This helps to refine the search for local waypoints and find the optimal path. The nonlinear inertia weight factor [29] is introduced to adaptively adjust the weights of the two algorithms and achieve a balance between the global exploration and local optimality search. The nonlinear weight factor update formula is shown in

(14) $$\omega =\omega_{\min}+(\omega_{\max}-\omega_{\min})\cdot \sin(\frac{\pi }{2}\cdot \frac{e}{e_{\max}}).$$

**Figure 1:**
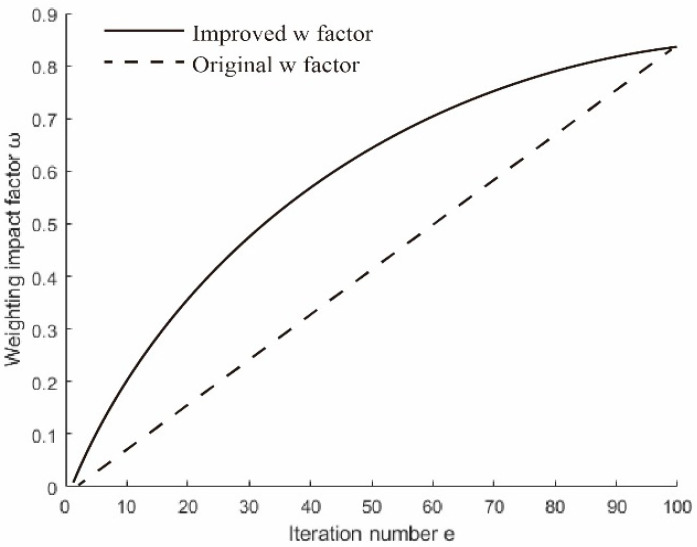
Comparison of adaptive weight factors.

The values of $\omega_{max}=$ 0.84 and $\omega_{min}=0.01$ are determined through extensive experiments. The traditional adaptive weight factor increases linearly, whereas the nonlinear weight factor employed here rises slowly in the early iterations, expanding the search range and thereby enhancing global exploration capability. In the later iterations, the increase rate of the weight factor accelerates, further strengthening the local exploitation capability. The variation in ω is illustrated in Figure 1.

An adaptive nonlinear inertia weight is incorporated into the position update mechanisms of both the swarm and beetle algorithms, as shown in

(15) $$c_{d,b}^{e+1}=\left\{\begin{array}{l} w\times c_{d,b}^{e}\exp(\frac{-b}{\alpha \cdot {\mathrm{dter}}_{\max}})\\ c{\mathrm{best}}_{b}^{e+1}+\frac{1}{B}\left(\sum_{b=1}^{B}\left|w\times c_{d,b}^{e}-c{\mathrm{best}}_{b}^{e+1}\right|\cdot \mathrm{rand}\{-1,1\}\right)\\ c_{d,b}^{e}+J(\frac{w\times c_{d,b}^{e}-c{\mathrm{worse}}_{b}^{e}}{\left|k_{d}-k_{w}\right|+\varepsilon }) \end{array}\right.,$$

(16) $$c_{b}^{e+1}=w\times c^{e}+{\mathrm{step}}^{e}\vec{d}\mathrm{sign}[k(c_{1}^{e})-k(c_{r}^{e})].$$

A dynamic coefficient ε is introduced to adjust the proportion of the two algorithms, regulate the ratio between current weights and weight changes from the previous generation during each weight update, and achieve a dynamic balance between global exploration and local exploitation through iterations, as shown in

(17) $$\left\{\begin{array}{l} c^{e+1}=(1-\varepsilon )c_{d,b}^{e+1}+\varepsilon c_{b}^{e+1}\\ \varepsilon =\frac{e}{2E} \end{array}\right..$$

### 2.4. Path Planning Based on IABCBAS Algorithm

**Figure 2:**
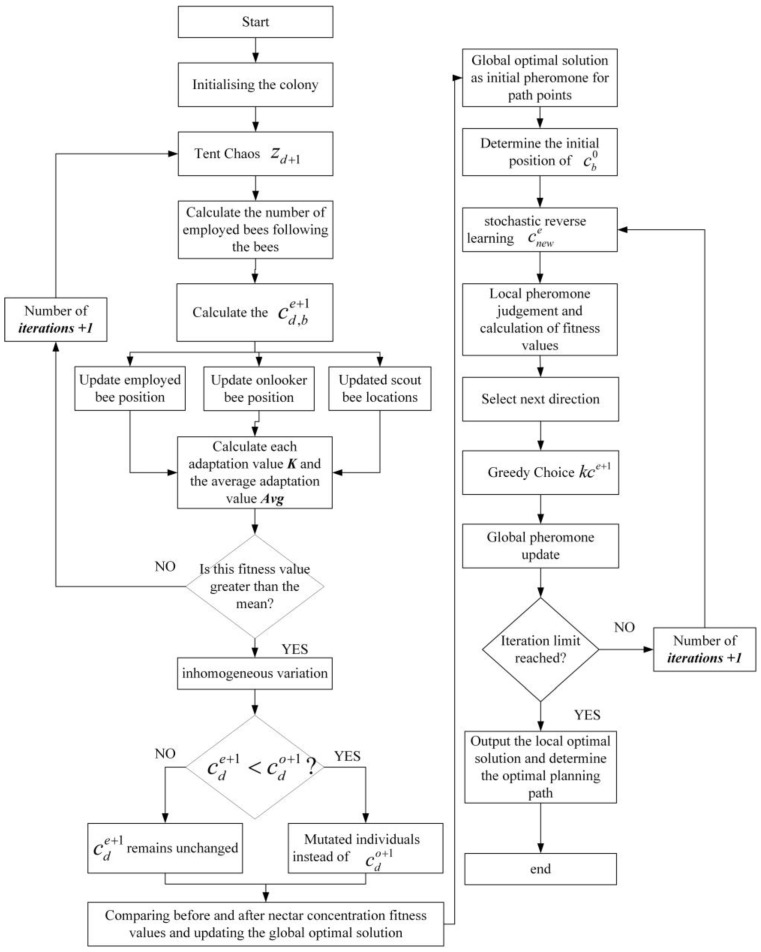
The flow path of the IABCBAS algorithm.

The path planning process based on the IABCBAS algorithm is illustrated in Figure 2, with the steps as follows:

(1) Set the relevant parameters for the ABC algorithm and generate the bee population with Tent chaotic perturbation using Equation (5).
(2) “Leading” and “following” are set as the main behaviors, interspersed with scouting, to form a new distribution of the honeybee population; obtain the corresponding nectar concentration, and select the colony distribution position based on nectar concentration.
(3) Mutate the bees after position update using Equations (6) and (7). If the nectar concentration after mutation is higher than that before mutation, update the position to the mutated one; otherwise, no operation is performed.
(4) If the maximum number of iterations of the bee colony algorithm is not reached, repeat the above cycle; otherwise, increase the initial pheromone value of the path points corresponding to the current bee colony to form an optimized initial pheromone distribution and improve the quality of global search points.
(5) Set the relevant parameters of the BAS algorithm and perform local numerical optimization using the optimized initial pheromone.
(6) Randomly generate a new solution using Equation (12) and calculate the fitness value of it. If the new solution outperforms the previous one, the direction of the optimization search is adjusted accordingly.
(7) The optimal solution is retained according to Equation (13), with local and global pheromones updated simultaneously.
(8) Check if the maximum number of iterations of the BAS algorithm has been reached. If so, the results of the optimal search are saved, analyzed, and processed.

### 2.5. Analysis of Algorithm Time Complexity

The time complexity of an algorithm can reflect its feasibility and efficiency in solving path planning problems. Therefore, this section conducts a time complexity analysis of the original ABC and BAS algorithms, as well as the improved IABCBAS algorithm.

In the global waypoint exploration phase, the size of the artificial bee colony is *C*, the problem dimension is *b*, and the maximum number of iterations of the algorithm is *E*. Therefore, the time complexity of the original ABC algorithm is $O\left(c\times b\times E\right)$. The initialized population of the improved ABC algorithm is disturbed by Tent chaos, and the initialization time complexity is $O\left(b\right)$. Affected by the mutation factor, the time complexity of the waypoint exploration phase is $O\left(c\times b\times E\right)$. Thus, the time complexity of the improved ABC algorithm phase is $O\left(b+c\times b\times E\right)$.

In the local waypoint optimization phase, the problem dimension at this stage is *b*. The computational complexity of initializing the beetle is $O\left(b\right)$. During the iteration process, the computational complexity for judging the directions of the two antennae is $O\left(2b\right)$, and the computational complexity for updating the beetle’s position is $O\left(2b\right)$. Therefore, the time complexity of the original BAS algorithm is $O\left(5b\right)$. Since the initial position of the beetle in the improved BAS algorithm is generated in the global exploration phase, the initialization computational complexity is reduced. Due to the introduction of random reverse solutions, the computational complexity caused by the increased direction selection is $O\left(3b\right)$. The computational complexity of greedy selection confirmation is $O\left(b\right)$. Thus, the time complexity of the improved BAS algorithm is $O\left(4b\right)$.

In summary, the combined time complexity of the two original algorithms before improvement is $O\left(c\times b\times E\right)+O\left(5b\right)=O\left(c\times b\times E+5b\right)$, and the complexity of the IABCBAS algorithm is $O\left(b+c\times b\times E\right)+O\left(4b\right)=O\left(c\times b\times E+5b\right)$. It can be seen that the IABCBAS algorithm has the same complexity of $O\left(c\times b\times E+5b\right)$. However, through the design of optimization strategies, the IABCBAS algorithm improves the search capability, controls the time, and improves the efficiency of path planning.

## 3. Experimental Testing of Search Performance on IABCBAS Algorithms

### 3.1. Test Functions and Environment

Four swarm intelligence algorithms from different periods are selected to conduct comparative experiments on nine test benchmark functions to verify the performance and feasibility of the proposed IABCBAS algorithms. All experiments are conducted on the same experimental platform. The comparison algorithms [16] and related parameters are shown in Table 1.

**Figure 3:**
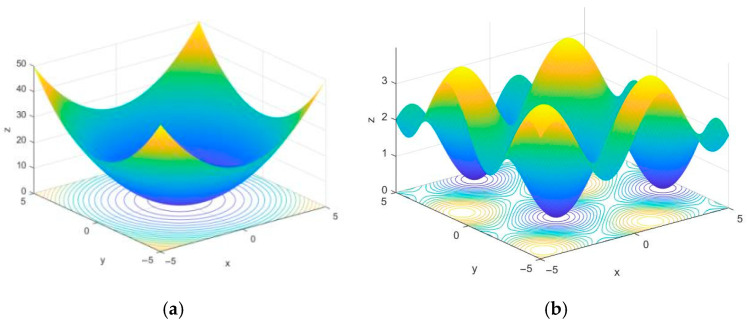
Benchmark test function modeling. (**a**) Sphere function; (**b**) Foxholes function.

Benchmark functions with different dimensions and numbers of peaks are employed to verify the algorithms’ convergence speed and their ability to escape local optima. The selected benchmark functions [30] are listed in Table 2, and part of the test function space is illustrated in Figure 3. The Sphere test function is a continuous unimodal convex function in a high-dimensional space with multiple local minima, enabling it to test the search algorithm’s ability to escape local minima. The Foxholes test function is a low-dimensional multimodal function with numerous canyons and obstacles, making the testing of algorithms more challenging.

### 3.2. Convergence and Stability Analysis

Performance comparison experiments are conducted on five algorithms and each benchmark function is run independently 50 times. The evaluation metrics include the best value, average value, and standard deviation. The population size is set to 40 and the maximum number of iterations is 500. The experimental results are presented in Table 3.

Among these functions, high-dimensional unimodal functions have only one global optimal point and no local extrema, which are mainly used to test convergence speed. Multimodal functions, which have multiple local extrema, are used to evaluate the performance of escaping local extrema across different dimensions (both high and low). For the three high-dimensional unimodal functions, the proposed IABCBAS algorithm achieves the theoretical optimal value of 0 when solving *f*_1_ and *f*_3_, with the corresponding standard deviation also being 0. In solving *f*_4_, the optimal value, average value, and standard deviation of the proposed IABCBAS algorithm are at least three orders of magnitude better than those of the other four algorithms.

Among the three high-dimensional multimodal functions, the WOA and SSA find the optimal values for $f_{4}$ and $f_{6}$, and the proposed IABCBAS algorithm maintains superior test performance; the proposed algorithm has much higher values for the $f_{5}$ function than that of the other four algorithms, reflecting improved search capability. For the three low-dimensional multimodal functions $f_{7}$ to $f_{9}$, our algorithm reaches the optimal value under the solution, demonstrating better optimization performance under low-dimensional conditions. In terms of stability, only the ACO, SSA, and our algorithm find the optimal solution for $f_{7}$, with their stability increasing in sequence. Our algorithm has the best stability for $f_{8}$ and ranks second only to the WOA for $f_{9}$. Across all algorithms, the accuracy on unimodal functions is higher than that on multimodal functions, and the accuracy on low-dimensional functions is higher than that on high-dimensional functions. The proposed IABCBAS algorithm achieves superior performance in both accuracy and stability. Regarding running time, different search mechanisms result in significant differences among algorithms. The average running times of the five algorithms are 3.994, 4.563, 5.597, 4.458, and 3.889 s, respectively. The proposed IABCBAS algorithm can generate multiple distinct solutions simultaneously, accelerating numerical comparisons and improving running time by 2.7% compared to the fastest PSO algorithm.

**Figure 4:**
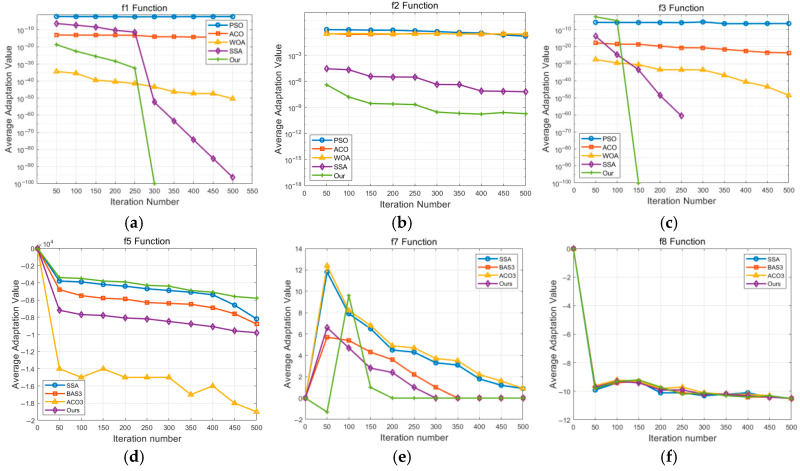
Comparison of benchmark function convergence curves. (**a**) Schwefel’s function; (**b**) Rosenbrock function; (**c**) Sphere function; (**d**) Schwefel function; (**e**) Foxholes function; (**f**) Schkel function.

The average convergence curves of the nine benchmark test functions are plotted to compare the convergence speed, accuracy, and ability to escape local optima among different algorithms, as shown in Figure 4. The vertical axis represents the fitness value, and the horizontal axis denotes the iteration number. Figure 4a–c demonstrate that the proposed IABCBAS algorithm exhibits faster convergence speed in unimodal functions, attributed to the chaotic initialization of the population. Simultaneously, the convergence accuracy of this algorithm significantly outperforms others. In both (a) and (c), the proposed algorithm escapes local extrema, whereas PSO, ACO, and WOA fail to do so. In the high-dimensional multimodal functions, ACO, WOA, SSA, and our algorithm achieve the optimal value of 0 in $f_{4}$ and $f_{6}$; thus, the convergence curves are not given due to early convergence. It is worth noting that our algorithm requires significantly fewer iterations than the other three algorithms and exhibits superior convergence performance. Figure 4d shows that the proposed IABCBAS algorithm falls into the local extreme value point in the 200th~300th generation, and there is a significant difference between the optimization value and the optimal value after 300 generations. However, the overall numerical curve amplitude is smaller and more stable than the other four algorithms. In the low-dimensional multimodal function, the numerical types of functions $f_{8}$ and $f_{9}$ are the same; only the former is shown in Figure 4f. All algorithms in Figure 4e,f converge to the optimal solution, but the proposed IABCBAS algorithm exhibits the fastest convergence speed.

### 3.3. Comparative Ablation Experiments

The proposed algorithm has been proven to exhibit superior global search capability and local optimization performance. However, additional experiments are required to verify (1) whether the selected mechanism is optimal, (2) whether it represents the optimal improvement for the proposed algorithm, and (3) whether the combination of the two algorithms is generalizable. To enhance the persuasiveness of model improvements, comparative algorithms are selected for experiments by reviewing the latest literature. The ABC algorithm is modified by introducing Bernoulli chaotic mapping, reverse learning mechanism, and KHO algorithm, respectively [29,30,31], denoted as ACO1-ACO3; the BAS algorithm is selected to combine with ABC, PSO, and AFSA [32,33,34], denoted as BAS1-BAS3. ACO1 and ACO2 were set up to compare with the Tent population Chaos and local random direction selection mechanism. ACO3 and BAS1-BAS3 were set up to compare the two algorithm-combining methods we used, respectively. The results are shown in Table 4.

It can be seen from Table 3 that compared with the original ABC algorithm, the added mechanisms have improved the algorithm’s optimality. The numerical results of ACO1 are all better than those of ACO2 in $f_{1}∼f_{6}$ function, indicating that enhancing population richness can effectively improve global search efficiency in high-dimensional functions. The numerical results of ACO2 are slightly better than that of ACO1 in function $f_{7}∼f_{9}$, indicating that increasing the random direction can increase local search stability in the low-dimensional function. Comprehensive results show that the results of each two combined algorithms are numerically better than the single-added mechanism results. The numerical results of BAS3 and ACO3 are better than those of BAS1 and BAS2, indicating that the combination of the new swarm intelligence algorithm has a wider search range and better enhancement effect. Meanwhile, the overall value of BAS1 is better than BAS2, indicating that the combination of the bee swarm and the beetle swarm is also good, which side by side verifies the accuracy of the basic algorithm selected. However, compared to the fish swarm and krill swarm algorithms, the beetle swarm algorithm has a simpler structure, giving the IABCBAS algorithm a greater advantage in search and optimization capabilities. Furthermore, taking the $f_{1}$ function as an example, the average value of the IABCBAS algorithm is the lowest among the eight algorithms, reaching $4.076\times 10^{-29}$, which indicates that the algorithm has a better optimization effect on the f1 function. Meanwhile, only the IABCBAS algorithm has a standard deviation of 0, suggesting that the results of multiple runs are highly stable without significant differences.

## 4. Simulation Experiment of IABCBAS Algorithm Path Planning Application

### 4.1. Establishment of Environmental Models

**Figure 5:**
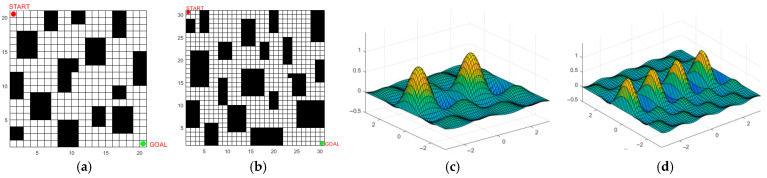
Establishment of environmental map model. (**a**) 20×20; (**b**) 30×30; (**c**) twin peaks terrain; (**d**) multimodal terrain.

The IABCBAS algorithm is capable of rapidly generating a large number of waypoints, conducting optimal path searches, and enabling mobile platforms to avoid obstacles in the environment through path planning, thereby enhancing the operational efficiency and environmental adaptability of the platforms. Particularly in path planning for field environments such as military operations, this algorithm can give full play to the advantages of search algorithms in solving high-dimensional space problems; it efficiently explores the optimal solutions for path planning in three-dimensional space and optimizes the kinematic constraints and performance constraints of mobile platforms. Therefore, the IABCBAS algorithm holds high application value in addressing path planning problems across multiple engineering application fields. Two-dimensional fixed-area and three-dimensional simulation map models are established based on different obstacle distributions in various environments to verify the universality of the proposed method, as shown in Figure 5. For the 2D environment, a simple map (20 × 20) and a complex map (30 × 30) are constructed, where obstacles are represented by black grids and free spaces by white grids. For the 3D environment, simple bimodal and complex multimodal elevation maps are built using elevation data.

In Figure 5a,b, the top left corner of the map is the starting point of the path planning and the bottom right corner is the endpoint. The fence side length is set as *B*, the fence space is divided into $M_{x}\;$ rows and $M_{y}$ columns with B unit length. The *n*-th fence grid coordinates are $\left(x_{n},y_{n}\right)$, and the formula is shown in

(18) $$\left\{\begin{array}{l} x_{n}=B\times \left(\left(i-1\right)\mathrm{mod}M_{x}\right)+\frac{B}{2}\\ y_{n}=B\times \left(M_{y}-\mathrm{ceil}\left(\frac{i}{M_{y}}\right)\right)+\frac{B}{2} \end{array}\right.,$$

where *mod* is the redundancy function and *ceil* is the upward rounding function.

**Figure 6:**
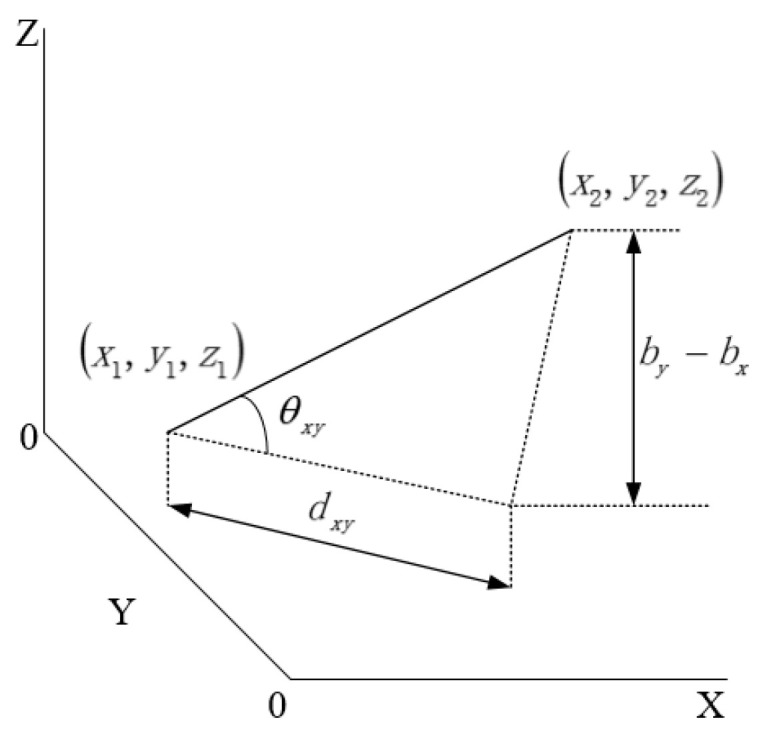
Schematic diagram of slope model.

The real coordinates are scaled down to one-tenth of their original values for the axes in Figure 5c,d to facilitate the 3D environment modeling. The slope model is generated based on the average slope between adjacent path points, as shown in Equation (19) and Figure 6.

(19) $$\theta_{1xy}=\mathrm{arc}\frac{b_{y}-b_{x}}{d_{xy}},$$

where *x* and *y* are the coordinates corresponding to adjacent elevation path points in the B-dimensional space, $\theta_{1xy}$ is the average slope of the path point $b_{y}$ relative to path point $b_{x}$, and $d_{xy}$ is the planar straight-line distance between the two path points.

### 4.2. Path Modeling

The path obtained by the search algorithm from the start point to the end point in the 2D grid map can be regarded as a set *T* of path points during motion planning. Path planning requires the vehicle to travel from the start point to the goal point, avoiding all obstacles and finding the shortest path, as shown in

(20) $$T=\left\{S,U_{1},U_{2},\ldots U_{M-1},G\right\},$$

(21) $$\mathrm{Length}T=\left\|U_{1}-S\right\|+\sum_{i=1}^{M-1}\left\|U_{i+1}-U_{i}\right\|+\left\|U_{M-1}-G\right\|,$$

(22) $$\left\{\begin{array}{l} T_{\min}=\arg\min\left({\mathrm{Length}}_{v}\right)\\ P\left({\mathrm{Length}}_{v}\right)=0 \end{array}\right.,$$

where *S* is the start point ($x_{0}$,$y_{0}$); $U_{1},U_{2},\ldots ,U_{M-1}$ is the via path node; *G* is the goal point ($x_{M}$,$y_{M}$); $T_{min}$ is the minimum value of the search path; ${Length}_{v}$ is the shortest planned path *v*; and $P(({Length}_{v})$ is the probability of collision between the path and the obstacle.

The path points are also treated as a series of discrete points in the 3D environment. The next movable path point is generated once the movement direction DIR is determined, as shown in Equation (23). The optimal path point that avoids obstacles is identified, as shown in Equation (24), and added to the shortest path $T_{min}Length$.

(23) $$\left\{\begin{array}{l} x_{i+1}=x_{i}+{\mathrm{dir}}_{x}\times \mathrm{rand}q_{1xy}\\ y_{i+1}=y_{i}+{\mathrm{dir}}_{y}\times \mathrm{rand}q_{1xy}\\ z_{i+1}=z_{i}+{\mathrm{dir}}_{z}\times \mathrm{rand}q_{1xy} \end{array}\right.,$$

(24) $$\left\{\begin{array}{l} U_{\min}=\sqrt{\left(U_{1}+U_{2}\right)\times \mathrm{pri}}\\ U_{1}=\sqrt{{\left(x_{i+1}-x_{i}\right)}^{2}+{\left(y_{i+1}-y_{i}\right)}^{2}+{\left(z_{i+1}-z_{i}\right)}^{2}}\\ U_{2}=\sqrt{{\left(x_{i}-x_{i-1}\right)}^{2}+{\left(y_{i}-y_{i-1}\right)}^{2}+{\left(z_{i}-z_{i-1}\right)}^{2}}\\ T_{\min}\mathrm{Length}=\sum_{i=0}^{B}u_{i} \end{array}\right.,$$

where $U_{1}$ and $U_{2}$ are the Euclidean distances between the current and the next path point and endpoint, respectively; and Pri is analogous to $P({Length}_{v})$, which avoids the searched path points colliding with obstacles in the map.

### 4.3. Comparison Experiment of Two-Dimensional Environment Simulation

In the initial stage, the improved ABC algorithm performs global search to generate multiple feasible paths, thereby enhancing the global search capability and verifying the effectiveness of path planning. The fitness value is calculated to determine the highest-quality global path, and the path points corresponding to the pheromone distribution are passed to the BAS algorithm. This reduces the blindness of the BAS algorithm’s search, strengthens its local optimization capability, and accelerates convergence.

**Figure 7:**
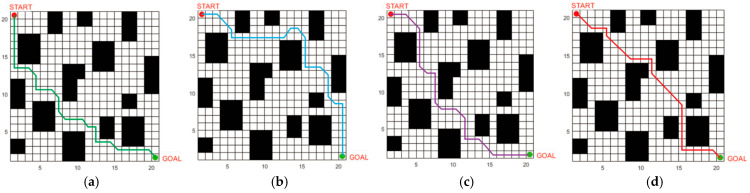
Optimal path planning results of four algorithms in a 20×20 2D simple environment: (**a**) SSA; (**b**) BAS3; (**c**) ACO3; (**d**) IABCBAS.

**Figure 8:**
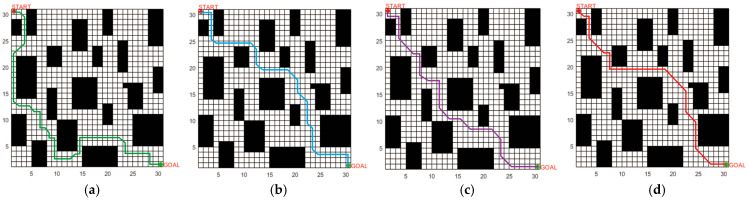
Optimal path planning results of four algorithms in a 30×30 2D complex environment: (**a**) SSA; (**b**) BAS3; (**c**) ACO3; (**d**) IABCBAS.

**Figure 9:**
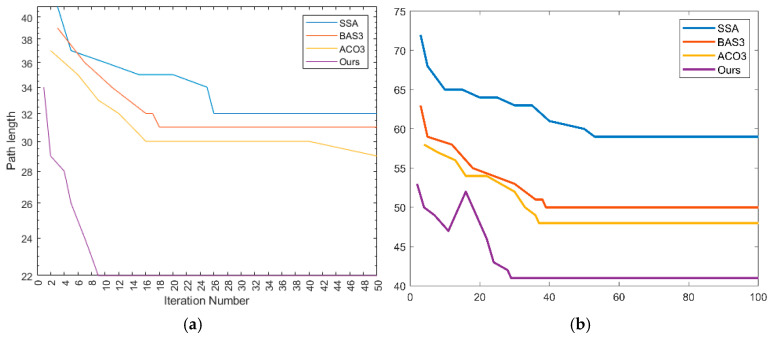
Convergence of four algorithmic paths in 2D environments: (**a**) 20×20 environmental convergence effect; (**b**) 30×30 environmental convergence effect.

The SSA, ACO3, and BAS3 algorithms are selected for comparative simulation experiments on path planning. Each algorithm runs 50 experiments in the fence map in Figure 5, with the following parameters: maximum iteration number = 500; search space dimension = 30; and population size = 100. All experiments are conducted under identical parameters. The path planning results in simple and complex environments are compared in Figure 7 and Figure 8, while the convergence comparison of different algorithms in these two environments is presented in Figure 9.

As observed in Figure 7 and Figure 9, for path planning in the 20 × 20 simple environment map, the SSA converges to the optimal solution at the 26th iteration, with an optimal path length of 32.4. However, the path contains numerous turns, which is unfavorable for the operation of unmanned vehicles in real environments. The BAS3 and ACO3 algorithms converge to the optimal solution successively at around the 18th and 17th iterations, with optimal path lengths of 29.8 and 26.4, respectively. Compared with the SSA, both exhibit fewer turns; however, their paths turn near obstacles, indicating weak space utilization capability. The proposed algorithm converges to the optimal solution at the ninth iteration, with a shorter planned path that is not affected by obstacles in space, thus achieving the best overall planning performance.

The comparison of the average values of the four algorithms over 500 iterations is presented in Table 5. For path planning in the complex environment map, the convergence times of the SSA, BAS3, and ACO3 algorithms increase with the number of obstacles, converging to the optimal solution at the 49th, 42nd, and 39th iterations, respectively. Additionally, the number of turns increases, and the path length becomes longer. The proposed IABCBAS algorithm yields an overall smoother path. Compared with SSA, BAS3, and ACO3, the number of turns is reduced by 46.32%, 28.74%, and 25.31%, respectively. Moreover, the planning time for the shortest path is 10.28 s faster than the average of the three algorithms. This indicates that the algorithm maintains superior planning performance in complex environments, meeting the current requirements of engineering applications.

### 4.4. Comparison Experiment of Three-Dimensional Environment Simulation

In this section, 3D path planning experiments are conducted on maps constructed from 3D elevation data to derive more comprehensive empirical conclusions and verify the adaptability of the proposed algorithm to real complex environments in 3D path planning. The comparison algorithms remain SSA, ACO3, and BAS3. For experimental data processing, the values in the simulation coordinates are multiplied by ten to convert them to real environment values. The experiment is repeated 30 times, and the average value of each index calculated by each algorithm is recorded to eliminate the interference of random errors. The experimental results are shown in Table 5.

**Figure 10:**
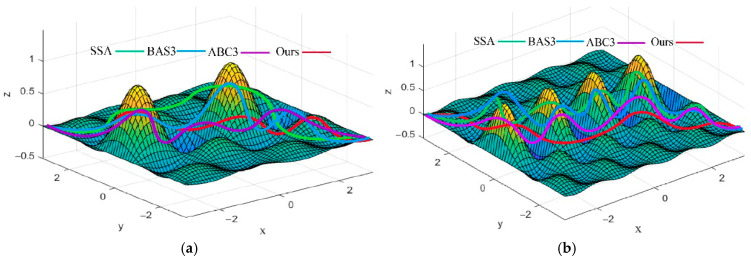
Optimal path planning results of four algorithms in the 3D environment: (**a**) twin peaks path planning results; (**b**) multimodal path planning results.

**Figure 11:**
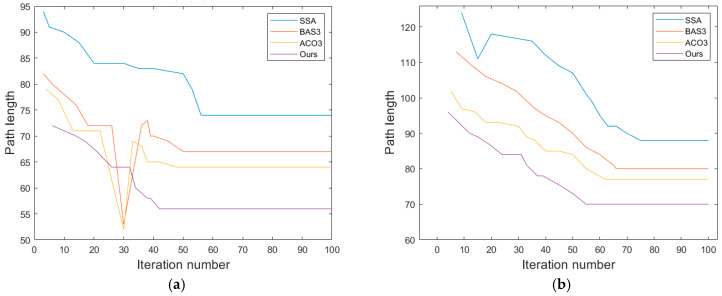
Convergence of 4 algorithmic paths in 3D environments: (**a**) convergence results for twin peaks environment; (**b**) convergence results for multimodal environment.

The results of optimal 3D path planning among different algorithms in the two environments are illustrated in Figure 10. The convergence comparison of different algorithms in these environments is presented in Figure 11. As can be seen from Figure 10 and Figure 11, when path planning in a 3D unimodal simple environment map, the SSA converges to the optimal solution around the 56th iteration, and the optimal path length is 74.1, which is 32.64% more than the proposed algorithm. The BAS3 and ACO algorithms converge to the optimal solution around the 50th and 48th generation, with optimal paths of 67.6 and 64.2, which are 18.73% and 13.57% longer than that of our method. The optimal paths are 67.6 and 64.2, respectively, which are 18.73% and 13.57% more than that of our method. Compared with the SSA, both algorithms exhibit fewer turns; however, their ability to traverse ascending and descending obstacles is weaker. The proposed IABCBAS algorithm converges to the optimal solution at the 42nd iteration, with a shorter planned path, superior obstacle-traversing capability, and the smoothest and most rational path in this environment.

As shown in Table 6 and Figure 10, for path planning in 3D multimodal complex environment maps, the number of iterations required for convergence increases with the number of obstacles for the SSA, BAS3, and ABC3 algorithms, which converge to the optimal solution around the 75th, 66th, and 62nd iterations, respectively. Additionally, their paths exhibit more turns, increased length, and reduced obstacle-traversing capability. In contrast, the path generated by the proposed IABCBAS algorithm is overall smooth. Compared with SSA, BAS3, and ABC3, the number of turns is reduced by 40.48%, 33.74%, and 26.65%, respectively. Moreover, the planning time for the shortest path is 13.62 s faster than the average of the three algorithms. These results indicate that the proposed IABCBAS algorithm maintains superior planning performance in complex environments, meeting the current requirements of real-world applications.

### 4.5. Comparison Experiment of Real Environment Simulation

**Figure 12:**
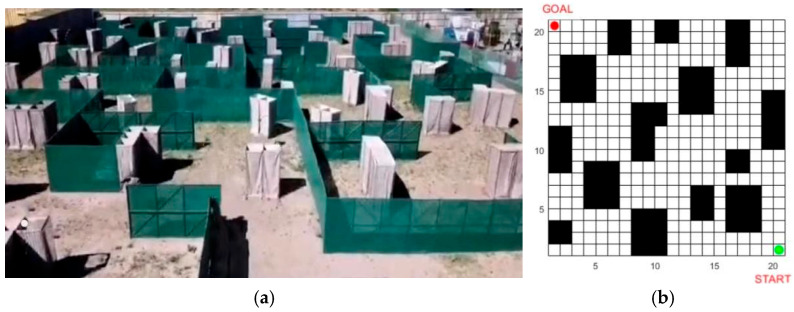
Real experimental environment and modeling: (**a**) real test environment; (**b**) real environment modeling.

An experimental site was selected for the path planning test of the mobile platform— specifically, a real environment containing obstacles—and the site environment was modeled. The model was invoked according to experimental requirements, and the real site and its environment modeling results are presented in Figure 12. In Figure 12a, the white obstacles in the target vehicle test site are represented by black square grids in the model. Additionally, the partitions in the target vehicle test site correspond to the grid boundary lines in the modeled map shown in Figure 12b.

**Figure 13:**
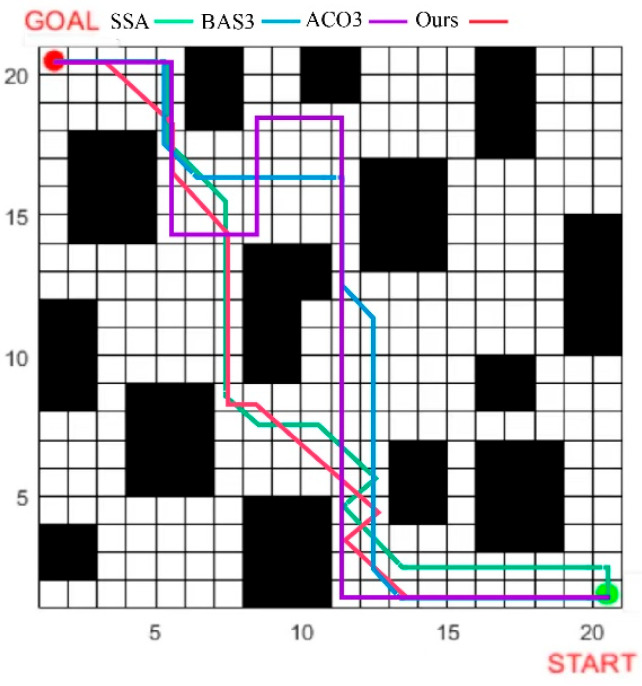
Route planning test results.

The mobile platform calls the algorithm to perform autonomous path planning driving in the test site, and the corresponding planned path is generated on the map by calling the modeling map during driving. The effect of each algorithm on path planning in the real environment is shown in Table 7 and Figure 13.

As presented in Table 7, our algorithm traverses the fewest grid squares in the simulation map and exhibits the optimal number of turns. The actual path distance is 92.6 m, which is 14.29%, 25.00%, and 35.71% shorter than those of the ACO3 (98.5 m), BAS3 (102.7 m), and SSA (108.3 m) algorithms, respectively. The planning time is 32.34 s, which is 16.14%, 24.86%, 31.14%, and 58.53% shorter than those of the four comparative algorithms, respectively. The proposed IABCBAS algorithm exhibits high efficiency in path planning for mobile target vehicles in real-world environments.

## 5. Conclusions

The IABCBAS algorithm is proposed to address the convergence and optimal search performance issues in swarm intelligence algorithms. Its search capability and stability are verified through tests on nine benchmark functions, with comparisons against four swarm intelligence algorithms from different periods. Ablation experiments are conducted to compare seven swarm intelligence algorithms with different optimization mechanisms, verifying the effectiveness of the introduced strategies in the improved algorithm. Application simulation results demonstrate that the proposed algorithm can rapidly plan a feasible path in the shortest time under environments of different dimensions. The proposed improvement mechanisms provide references for the optimization and enhancement of swarm intelligence algorithms, as well as their applications in other engineering fields. In future work, path planning can be integrated with curve processing strategies to further enhance path smoothness and planning accuracy. Additionally, the curvature optimization of inflection points can be improved to adapt to more complex real-world terrain environments.

## Figures and Tables

**Table 1:** The comparison algorithms and related parameters.

Algorithm	Parameter Setting	Parameter Meaning
PSO	$c_{1}=c_{2}=2$ $\omega \in \left[0.5,0.8\right]$	$c_{1},\;c_{2}$ are learning factor, ω is inertia weight, adjustment of particle speed
ACO	α=1 β=3 p=0.6	α is pheromone heuristic factor, β is heuristic function factor, *p* is pheromone evaporation coefficient
SSA	$P_{dp}=0.2$ $G_{c}=1.8$ sf=18	$P_{dp}$ is sparrows exploring new areas, $G_{c}$ is sparrows that warn of danger, *sf* is alert threshold
WOA	$\alpha_{s}=0.15$ ε=0.01 b=2	$\alpha_{s}$ is linear control parameter, ε is hunting probability factor, *b* is bubble spiral sparsity

**Table 2:** The nine benchmark parameters for algorithm testing.

**Type**	**Parameter**	**Basis Function**	Function	Dimension	Corridor	Optimum Value
Higher dimensional unimodal function	$f_{1}$	Schwefel’s	$f_{1}(x)=\sum_{i=1}^{N}\left|x_{i}\right|+\prod_{i=1}^{N}\left|x_{i}\right|$	30	[−10,10]	0
	$f_{2}$	Rosenbrock	$f_{2}(x)=\sum_{i=1}^{N-1}\left[100{\left(x_{i+1}-x_{i}^{2}\right)}^{2}+{\left(x_{i}-1\right)}^{2}\right]$	30	[−30,30]	0
	$f_{3}$	Sphere	$f_{3}(x)=\sum_{i=1}^{N}x_{i}^{2}$	30	[−100,100]	0
Higher dimensional multimodal function	$f_{4}$	Rastrigrin	$f_{4}(x)=\sum_{i=1}^{N-1}\left[x_{i}^{2}-10\cos\left(2\pi x_{i}\right)+10\right]$	30	[−5.12,5.12]	0
	$f_{5}$	Schwefel	$f_{5}(x)=\sum_{i=1}^{N}-x_{i}\sin\left(\sqrt{\left|x_{i}\right|}\right)$	30	[−500,500]	−418.98
	$f_{6}$	Griewing	$f_{6}(x)=\frac{1}{4000}\sum_{i=1}^{N}x_{i}^{2}-\prod_{i=1}^{N}cos(\frac{x_{i}}{\sqrt{i}})+1$	30	[−600,600]	0
Low-dimensional multi-maximilian function	$f_{7}$	Foxholes	$f_{7}(x)={\left(\frac{1}{500}+\sum_{j=1}^{25}\frac{1}{j+\sum_{i=1}^{2}{\left(x_{i}-a_{ij}\right)}^{6}}\right)}^{-1}$	2	[−65,65]	1
	$f_{8}$	Schkel	$f_{8}(x)=-\sum_{i=1}^{10}{\left[(X-a_{i}){\left(X-a_{i}\right)}^{T}+c_{i}\right]}^{-1}$	4	[−10.54,10.54]	−10.54
	$f_{9}$	Hartmann 6-D	$f_{9}(x)=-\sum_{i=1}^{4}c_{i}exp\left(-\sum_{j}^{6}a_{ij}{\left(x_{j}-p_{ij}\right)}^{2}\right)$	6	[−3.52,0]	−3.32

**Table 3:** Comparison of benchmark function test results.

**Norm**	**Algorithms**	$f_{1}$	$f_{2}$	$f_{3}$	$f_{4}$	$f_{5}$	$f_{6}$	$f_{7}$	$f_{8}$	$f_{9}$
Optimal value	PSO	$5.684\times 10^{-4}$	$1.635\times 10^{-1}$	$1.527\times 10^{-7}$	2.906	$-8.207\times 10^{3}$	$2.453\times 10^{-7}$	0.8909	−10.54	−3.322
	ACO	$3.291\times 10^{-15}$	$2.661\times 10^{-1}$	$3.206\times 10^{-24}$	0	$-8.756\times 10^{3}$	0	1	−10.54	−3.322
	WOA	$4.672\times 10^{-51}$	$2.808\times 10^{-1}$	$3.579\times 10^{-49}$	0	$-1.972\times 10^{4}$	0	0.8909	−10.54	−3.322
	SSA	$1.738\times 10^{-97}$	$6.431\times 10^{-8}$	0	0	$-9.816\times 10^{3}$	0	1	−10.54	−3.322
	Our	0	$1.856\times 10^{-11}$	0	0	$-5.839\times 10^{3}$	0	1	−10.54	−3.322
Average value	PSO	$4.915\times 10^{-3}$	$6.159\times 10^{-1}$	$1.822\times 10^{-6}$	5.206	$-5.106\times 10^{3}$	$1.731\times 10^{-2}$	4.536	−9.561	−3.472
	ACO	$7.693\times 10^{-15}$	$2.993\times 10^{-1}$	$2.255\times 10^{-21}$	3.714	$-6.658\times 10^{3}$	$3.545\times 10^{-3}$	3.729	−10.33	−3.452
	WOA	$4.798\times 10^{-42}$	$3.097\times 10^{-1}$	$5.304\times 10^{-34}$	0	$-1.507\times 10^{4}$	0	4.883	−7.911	−3.409
	SSA	$1.659\times 10^{-11}$	$4.391\times 10^{-6}$	$2.727\times 10^{-61}$	0	$-8.584\times 10^{3}$	0	3.487	−8.805	−3.482
	Our	$3.148\times 10^{-26}$	$2.178\times 10^{-9}$	0	0	$-4.541\times 10^{3}$	0	3.119	−10.006	−3.421
Standard deviation	PSO	$6.161\times 10^{-3}$	$5.179\times 10^{-1}$	$2.301\times 10^{-6}$	1.964	$1.801\times 10^{3}$	$9.233\times 10^{-4}$	5.196	2.508	$5.902\times 10^{-2}$
	ACO	$4.908\times 10^{-15}$	$5.476\times 10^{-1}$	$2.899\times 10^{-21}$	4.241	$9.407\times 10^{2}$	$8.105\times 10^{-4}$	4.184	1.382	$8.541\times 10^{-2}$
	WOA	$7.056\times 10^{-42}$	$4.426\times 10^{-1}$	$4.375\times 10^{-33}$	0	$1.679\times 10^{3}$	0	5.125	3.683	$1.185\times 10^{-1}$
	SSA	$3.851\times 10^{-12}$	$2.414\times 10^{-5}$	$3.163\times 10^{-61}$	0	$5.154\times 10^{2}$	0	3.422	2.256	$5.741\times 10^{-2}$
	Our	0	$3.707\times 10^{-8}$	0	0	$4.724\times 10^{3}$	0	2.692	$3.086\times 10^{-11}$	$3.411\times 10^{-2}$
Running time	PSO	4.691	4.006	3.821	4.225	3.712	3.731	4.115	3.301	4.347
	ACO	5.155	4.802	4.963	4.821	4.267	4.446	4.297	3.633	4.679
	WOA	8.403	6.308	5.889	5.563	5.953	5.274	4.708	4.037	4.239
	SSA	5.323	4.702	4.521	4.227	4.832	4.389	3.813	3.691	4.621
	Our	4.469	4.128	3.689	4.043	3.406	3.385	3.729	3.415	4.386

**Table 4:** Test results of each algorithm on the benchmark function.

Algorithm	$f_{1}$ Function		$f_{2}$ Function		$f_{3}$ Function	
	Mean	Std	Mean	Std	Mean	Std
ABC	$3.243\times 10^{-9}$	$5.003\times 10^{-8}$	$3.132\times 10^{-1}$	$4.233\times 10^{-1}$	$4.431\times 10^{-4}$	$3.165\times 10^{-3}$
ACO1	$1.605\times 10^{-20}$	$2.327\times 10^{-19}$	$4.292\times 10^{-2}$	$3.482\times 10^{-2}$	$2.076\times 10^{-8}$	$1.297\times 10^{-7}$
ACO2	$1.763\times 10^{-17}$	$2.301\times 10^{-16}$	$3.914\times 10^{-3}$	$2.631\times 10^{-2}$	$2.435\times 10^{-8}$	$1.371\times 10^{-7}$
ACO3	$3.363\times 10^{-27}$	$2.092\times 10^{-26}$	$2.472\times 10^{-11}$	$4.972\times 10^{-10}$	$6.316\times 10^{-31}$	$2.124\times 10^{-30}$
BAS1	$1.026\times 10^{-22}$	$1.924\times 10^{-21}$	$5.594\times 10^{-7}$	$2.741\times 10^{-6}$	$4.291\times 10^{-19}$	$1.137\times 10^{-18}$
BAS2	$5.084\times 10^{-21}$	$5.221\times 10^{-20}$	$6.357\times 10^{-6}$	$4.738\times 10^{-5}$	$4.578\times 10^{-18}$	$1.119\times 10^{-17}$
BAS3	$5.675\times 10^{-26}$	$3.929\times 10^{-25}$	$4.771\times 10^{-10}$	$3.829\times 10^{-9}$	$5.581\times 10^{-24}$	$2.186\times 10^{-23}$
IABCBAS	$4.076\times 10^{-29}$	0	$3.122\times 10^{-13}$	$2.891\times 10^{-12}$	0	0
**Algorithm**	**$f_{4}$ function**		**$f_{5}$ function**		**$f_{6}$ function**	
	**Mean**	**Std**	**Mean**	**Std**	**Mean**	**Std**
ABC	2.698	1.627	$-3.133\times 10^{2}$	$3.156\times 10^{2}$	$1.957\times 10^{-2}$	$6.452\times 10^{-4}$
ACO1	2.063	1.614	$-4.497\times 10^{2}$	$3.661\times 10^{2}$	$1.204\times 10^{-2}$	$7.442\times 10^{-4}$
ACO2	2.274	2.014	$-4.398\times 10^{2}$	$3.349\times 10^{2}$	$1.705\times 10^{-2}$	$7.452\times 10^{-4}$
ACO3	0	0	$-8.367\times 10^{2}$	$8.427\times 10^{2}$	0	0
BAS1	0	0	$-6.823\times 10^{2}$	$5.391\times 10^{2}$	0	0
BAS2	0	0	$-6.332\times 10^{2}$	$5.665\times 10^{2}$	0	0
BAS3	0	0	$-7.422\times 10^{2}$	$7.308\times 10^{2}$	0	0
IABCBAS	0	0	$-5.282\times 10^{3}$	$5.511\times 10^{3}$	0	0
**Algorithm**	**$f_{7}$ function**		**$f_{8}$ function**		**$f_{9}$ function**	
	**Mean**	**Std**	**Mean**	**Std**	**Mean**	**Std**
ABC	5.317	4.223	−7.868	2.718	−3.224	0.0596
ACO1	5.479	4.604	−6.312	3.117	−3.168	0.0617
ACO2	5.328	4.535	−6.653	3.129	−3.172	0.0612
ACO3	4.263	3.557	−9.938	1.219	−3.396	0.0432
BAS1	4.812	4.134	−8.362	2.346	−3.254	0.0569
BAS2	4.766	4.117	−8.504	2.512	−3.298	0.0584
BAS3	4.339	3.692	−9.556	1.532	−3.387	0.0479
IABCBAS	3.218	2.692	−10.006	0.825	−3.407	0.0363

**Table 5:** Comparison of average performance of four algorithms in two-dimensional environments.

Environments	Method	Average Path Length (m)	Average Number of Turns	Average Number of Iterations	Average Planning Time (s)
20×20 2D simple environment	SSA	34.8	16.3	28.5	15.58
	BAS3	31.4	13.2	21.2	11.59
	ACO3	29.7	12.5	19.7	10.77
	IABCBAS	26.2	9.8	11.1	6.07
30×30 2D complex environment	SSA	62.6	23.1	49.3	39.28
	BAS3	52.9	17.4	41.8	33.31
	ACO3	50.5	16.6	39.4	31.39
	IABCBAS	44.3	12.4	30.6	24.38

**Table 6:** Comparison of average performance of four algorithms in three-dimensional environments.

Environments	Method	Average Path Length (m)	Average Numberof Turns	Average Numberof Iterations	Average Planning Time (s)
Three-dimensional unimodal simplex environment	SSA	77.2	35.2	58.5	52.76
	BAS3	69.1	30.1	52.6	44.36
	ACO3	66.1	27.4	50.6	41.38
	IABCBAS	58.2	21.5	44.8	36.37
Three-dimensional multimodal complex environments	SSA	91.1	54.1	77.4	68.12
	BAS3	82.4	48.6	68.2	58.91
	ACO3	79.4	43.9	65.7	55.85
	IABCBAS	72.1	32.2	57.2	47.34

**Table 7:** Comparison of average performance of four algorithms in real environments.

Environments	Method	Average Path Length (m)	Average Number of Turns	Average Number of Iterations	Average Planning Time (s)
Real environment	SSA	108.3	65.9	90.3	51.27
	BAS3	102.7	60.1	84.7	42.41
	ACO3	98.5	57.4	80.5	40.38
	IABCBAS	92.6	50.8	72.1	32.34

## Data Availability

The original contributions presented in this study are included in the article. Further inquiries can be directed to the corresponding authors.
